# Correlations between coagulation abnormalities and inflammatory markers in trauma-induced coagulopathy

**DOI:** 10.3389/fphys.2024.1474707

**Published:** 2024-10-30

**Authors:** Ke Wen, Zhexuan Lin, Haizhu Tan, Ming Han

**Affiliations:** ^1^ Department of Hand and Microsurgery, Taihe Hospital, Affiliated Hospital of Hubei University of Medicine, Shiyan, Hubei, China; ^2^ Bio-Analytical Laboratory of Shantou University Medical College, Shantou, China; ^3^ Department of Preventive Medicine, Shantou University Medical College, Shantou, China; ^4^ Emergency Department of Shenzhen University General Hospital, Shenzhen, China

**Keywords:** trauma-induced coagulopathy, tissue damage, coagulation dysfunction, monitoring of physiological indicators, multiple traumas model

## Abstract

**Introduction:**

In multiple trauma patients, the occurrence of trauma-induced coagulopathy (TIC) is closely associated with tissue damage and coagulation function abnormalities in the pathophysiological process.

**Methods:**

This study established a multiple trauma and shock model in Sprague-Dawley (SD) rats and comprehensively utilized histological staining and radiographic imaging techniques to observe injuries in the intestine, liver, skeletal muscles, and bones. Monitoring activated partial thromboplastin time (APTT), platelet (PLT) count, respiratory rate, blood pressure, and other physiological indicators revealed time-dependent alterations in coagulation function and physiological indicators. Enzyme-linked immunosorbent assay (ELISA) measurements of inflammatory factors Tumor Necrosis Factor-alpha (TNF-α), Interleukin-6 (IL-6) and vascular endothelial injury marker (Syndecan-1) were also conducted.

**Results:**

Experimental results demonstrated significant changes in tissue structure after multiple traumas, although widespread necrosis or hemorrhagic lesions were not observed. There were time-dependent alterations in coagulation function and physiological indicators. ELISA measurements showed a strong positive correlation between the significant decrease in PLT count and the increase in TNF-α and IL-6 concentrations.

**Discussion:**

The study provides crucial information for the early diagnosis and treatment of TIC. The findings suggest that structured monitoring of coagulation and inflammatory indicators can help in understanding the pathophysiological changes and aid in the management of TIC in multiple trauma patients.

## Introduction

In the fields of clinical emergency and critical care medicine, trauma-induced coagulopathy (TIC) is a common complication that significantly impacts the treatment outcomes and prognosis of multiple trauma patients (Horst et al., 2020). TIC is often accompanied by significant bleeding, tissue damage, and inflammatory responses, involving a complex pathophysiological process with multiple metabolic pathways and regulatory networks (Blaine and Steurer, 2019). Epidemiological studies have shown that TIC not only increases the risk of mortality in trauma patients but also escalates the use of healthcare resources and medical costs (Meizoso et al., 2022; Zipperle et al., 2022). Therefore, effective prevention and treatment of TIC are crucial for improving patient survival rates.

The pathogenesis of TIC is highly complex, involving immediate dysfunction of coagulation function post-injury, excessive release of inflammatory mediators, and activation of the fibrinolytic system, among other biological processes (Sastry et al., 2022; Iba et al., 2020; Levi, 2021). These processes interact, leading to microvascular dysfunction, increased thrombosis, and a predisposition to bleeding (Katneni et al., 2020). Some progress has been made in the study of biological markers for TIC, such as prolonged prothrombin time (PT) and decreased platelet (PLT) count, but these markers often manifest in the late stages of the disease and offer limited assistance for early intervention and prognosis evaluation (Dempsey et al., 2021; Loh et al., 2021; Cave et al., 2022).

In the study of TIC, animal models are effective tools for understanding its pathogenesis and evaluating therapeutic approaches (Hsu et al., 2021; Li et al., 2021; Machida, 2020). By mimicking the biological responses following human trauma, animal models can assist us in investigating the occurrence and progression of TIC at the molecular, cellular, and systemic levels. However, currently utilized animal models, such as the Rat model, still face challenges regarding accuracy and reproducibility in simulating human TIC (Li and Zhang, 2021; Carter et al., 2020; Zhang et al., 2021). For instance, the physiological differences between small animals and humans may influence the generalizability of research findings (Liu et al., 2022).

Despite existing studies providing valuable insights into tissue damage post multiple traumas, there remains insufficient understanding of tissue damage under the combined impact of shock and coagulation function disorders. Histological staining and radiological imaging are currently common methods for assessing TIC (Kajiwara et al., 2023). Histological staining can visually display changes in tissue structure, while radiological imaging can non-invasively monitor fractures and visceral injuries (Song et al., 2022; Zhang et al., 2023; Zheng et al., 2022). However, these methods have limitations in evaluating coagulation function disorders and physiological indicator changes, such as their inability to provide real-time and continuous biological information, restricting their applicability in animal models (Best et al., 2019; Ghayad and Barakett-Hamadé, 2022; Rawn et al., 2022).

This study is the first to reveal detailed tissue structural changes in a rat model of multiple trauma, systematically demonstrating the microstructural damage to the rat intestine and liver after trauma, providing extensive histological evidence. We also found that changes in activated partial thromboplastin time (APTT) and platelet count exhibited significant time dependence, and confirmed the continuous decline in rat respiratory rate and blood pressure. These findings offer new, such as biomarkers Tumor Necrosis Factor-alpha (TNF-α) and Interleukin-6 (IL-6), for the early diagnosis and intervention strategies of trauma-induced coagulopathy, providing a theoretical basis and practical guidance for future clinical applications.

Given the background outlined above, this study constructed a Rat model of multiple traumas and shock and comprehensively evaluated post-traumatic tissue damage, coagulation function, and inflammatory responses by employing histological staining and radiological imaging techniques combined with monitoring of physiological indicators. Continuously monitoring key physiological parameters such as activated partial thromboplastin time (APTT), PLT count, respiratory rate, and blood pressure, along with assessing fibrinogen (Fib), D-dimer levels, inflammatory cytokines (TNF-α and IL-6), and endothelial injury marker (Syndecan-1), aims to identify new biomarkers for TIC and explore their time-dependent characteristics. The objectives of this study are to provide novel theoretical foundations and practical strategies for early diagnosis and intervention of TIC, particularly in the management of TIC under conditions of multiple traumas and shock. The findings of this research contribute to a deeper understanding of the pathogenesis of TIC but also have the potential for positive impacts on the practice of clinical emergency care and critical care medicine.

## Materials and methods

### Establishment of TIC model in Sprague-Dawley (SD) rats

This study was approved by the Ethics Committee of Shantou University Medical College, with the approval number SUMC 2018-127. All experiments were conducted strictly according to the Animal Care and Use Guidelines of the Experimental Animal Center. SD rats used in the experiment were sourced from the Animal Center of Shantou University Medical College. The rats were housed under appropriate temperature and humidity conditions, provided with 15 g of feed per 100 g of body weight, and had continuous access to water.

Prior to the experiment, the rats were fasted for 12 h but allowed free access to water. Subsequently, anesthesia was induced by intraperitoneal injection of 2% pentobarbital sodium, and surgical procedures were carried out to open the airway. Under sterile conditions, the rats’ left femoral artery and left carotid artery were dissected, followed by the insertion of catheters after ligating the distal end of the femoral artery and clamping the proximal end. The femoral artery catheter was connected to a T-tube for continuous blood sampling. Heparin saline was infused into the carotid artery catheter to monitor mean arterial pressure simultaneously.

Male SD rats weighing 350–400 g were randomly divided into an uninjured control group (n = 10) and a Multiple traumas group (n = 10). In the Multiple traumas model group, in addition to the steps above, additional trauma induction was performed. Specifically, after disinfection with iodine, abdominal surgery was conducted for access, involving a midline incision. A 10 cm segment of the small intestine anterior to the cecum was isolated, and moderate to severe compression injury was induced gently using forceps. Repetitive compression injuries were applied to the right leg muscle and liver lobes with the same forceps, followed by suturing the abdominal incision. Subsequently, 6 stainless steel balls weighing 65 g were dropped from 36 inches through a catheter using a modified three-point impact device. The right leg skeletal muscles were compressed 10 times, resulting in fractures of the right tibia and fibula in the rats. Finally, blood was withdrawn through the femoral artery catheter to lower the mean arterial pressure to 40 mmHg within 5 min and maintained at 40% removal of estimated blood volume. Bleeding was typically completed within 30 min to assess coagulation parameters and PLT count. Free recovery of blood pressure and respiration was halted. Subsequently, all groups were monitored for 150 min without further intervention. At the end of the experiment, collected tissues were subjected to hematoxylin and eosin (HE) and Masson staining, as well as routine radiological observation of tibial and fibular fractures.

### Organizational and radiological assessment methods

After the experiment, tissue samples of rats’ liver, small intestine, and skeletal muscle were collected. These samples were initially fixed with 10% formalin, followed by dehydration in increasing ethanol concentrations (70%, 80%, 90%, 95%, 100%), and embedded in paraffin. Subsequently, 4 μm thick tissue sections were cut, deparaffinized in xylene for 10 min, repeated twice, sequentially immersed in 100%, 95%, 80%, and 70% ethanol for 2 min each, and rinsed with distilled water. The sections were stained using hematoxylin for 10 min and eosin for 2 min (H&E staining) to evaluate cell morphology changes and pathological status. Masson’s trichrome staining was then performed by staining the sections with Weigert’s iron hematoxylin for 10 min, followed by staining with Ponceau-fuchsin solution for 5 min, differentiation in phosphomolybdic acid for 10 min, staining with aniline blue solution for 5 min, differentiation in 1% acetic acid for 2 min, final rinsing in water, dehydration, and mounting.

Subsequently, the stained sections were observed under an optical microscope (Olympus BX50, Tokyo, Japan) to meticulously record the morphological characteristics of the injury site. The liver tissue sections were scored for injury using the liver injury scoring system developed by Suzuki et al.: 0 for no injury, 1 for mild injury characterized by slight hepatocyte swelling without hemorrhage, 2 for moderate injury with noticeable hepatocyte swelling and localized bleeding, 3 for severe injury including hepatocyte necrosis and extensive bleeding, and 4 for very severe injury presenting extensive hepatocyte necrosis and severe bleeding. Intestinal tissue scoring was based on the Nadler criteria: 0 for normal intestinal mucosal villi, 1 for mild separation of mucosal or submucosal layers, 2 for moderate separation of mucosal or submucosal layers or edema between submucosal and muscular layers, 3 for severe separation of mucosal or submucosal layers or severe edema between submucosal and muscular layers, 4 for full-thickness intestinal wall necrosis; a score of ≥2 indicates NEC positivity. Skeletal muscle tissue scoring followed Gallet’s muscle injury grading system: 0 for no damage with orderly arranged muscle fibers, no significant cell swelling or necrosis, and no bleeding, 1 for mild injury with slight muscle fiber swelling and minimal bleeding, 2 for moderate injury with moderate muscle fiber swelling and evident bleeding, 3 for severe injury with extensive muscle fiber swelling or necrosis and widespread bleeding, and 4 for extremely severe injury with disrupted muscle fiber structure, severe necrosis, and heavy bleeding.

Additionally, post-fracture operations on rats were subjected to conventional radiological examination, where X-rays of the tibia and fibula were taken using a Direct Radiography (DR) system in both anterior-posterior and lateral views to observe and document the location, type, and severity of the fracture, aiding in a more accurate evaluation of the multiple trauma model’s effectiveness.

To ensure the objectivity of the data and minimize researcher bias, this study employed an independent blind assessment. All histological samples were evaluated by external pathologists who were not involved in the experimental design or execution. Before evaluation, all samples were randomized and coded to ensure blinding in the assessment. Pathologists scored the samples based on pre-established standardized scoring systems, and the scoring results were used for subsequent statistical analyses.

### Blood sample collection and analysis methods

In this study, the initial blood sample collection in rats was performed post-anesthesia before inducing multiple traumas and hemorrhagic shock (time point 0), serving as self-control. Subsequently, additional blood samples were collected at 30, 60, 90, 120, and 150 min post hemorrhagic shock induction. The initial blood volume collected was 630 μL, with the addition of 70 μL of 3.2% sodium citrate as an anticoagulant. All blood samples were centrifuged at 2000×g for 10 min to collect the supernatant for analyzing PT, APTT, International Normalized Ratio (INR), Fib, and D-dimer levels using the Sysmex CA-7000 automated coagulation analyzer. PLT count was determined microscopically after treatment with potassium EDTA. The second blood collection volume was 1 mL, immediately transferred to a tube without an anticoagulant, left to stand at room temperature for 30 min, and centrifuged at 3,000 × g for 15 min to separate serum. The collected serum was promptly transferred to a −80°C freezer for subsequent biochemical analysis. Using reagent kits provided by the American R&D company, TNF-α, IL-6 concentrations, and Syndecan-1 levels in rat plasma were measured by enzyme-linked immunosorbent assay (ELISA) following the kit instructions. The experiment ended at 150 min, at which point euthanasia was performed on the animals. Liver, small intestine, and right leg skeletal muscle tissues were then collected for future histological examination (Table 1).

**TABLE 1:** Heterogeneity analysis of initial APTT, platelet count, breath rate, and blood pressure parameters.

	*p*-value
APTT	0.28
Plt	0.06
Breath rate	0.41
Blood pressure	0.14
Fib	0.22
D-dimer	0.08
TNF-α	0.07
IL-6	0.06
Syndecan-1	0.42

### Statistical analysis methods

This study primarily utilized SPSS software (version 26.0, IBM Corp., Armonk, NY, USA) for statistical processing, and numerical data were expressed as mean ± standard deviation (Mean ± SD). The paired Wilcoxon test was employed to analyze the data of the Multiple traumas group at different time points (30, 60, 90, 120, 150 min) compared to baseline (0 min) to investigate the changes in coagulation indicators, inflammatory factors, and vital signs over time. Spearman’s rank correlation was applied to explore the correlation between PLT count after Multiple traumas and inflammatory factors (TNF-α and IL-6). A significance level of 0.05 was set. Finally, line trend graphs and box plots were used to describe the trends of APTT and PLT over time to identify potential peak points throughout the process.

## Results

### Observation of tissue damage after multiple traumas and shock

Multiple trauma and shock are clinically significant syndromes that pose a serious threat to life. Their complex pathological mechanisms involve damage responses in multiple organs and systems. This study aimed to provide theoretical support for clinical treatment by investigating the effects of multiple trauma and shock on the liver, small intestine, and skeletal muscle. A specially designed atraumatic vascular clamp was used to induce crush injuries in these organs in rats, followed by detailed pathological observations post-injury.

By placing a circular clamp approximately 1.5 cm from the edge of the liver lobe, we induced crush injuries in the liver. It resulted in subcapsular hemorrhage and hematoma, as well as subcapsular liver lacerations. Three lines of hematomas were observed in the middle and right liver lobes, but no significant intra-abdominal bleeding was noted. After the small intestine was subjected to crush injury, slight bleeding was observed, specifically manifesting as mesenteric ischemia, congestion, edema, and contusions of the intestinal wall. The crush injury led to vascular dilation, congestion, accompanied by edema and hemorrhagic spots in the intestinal wall.

Following the crush injury to the skeletal muscle, the injured area exhibited significant edema, with slight bleeding on the muscle surface, accompanied by contusions of muscle fibers. In our model, no massive uncontrolled bleeding was observed (Supplementary Figure S1).

### Structural changes in rat tissues of intestine, liver, and skeletal muscle after multiple traumas and shock: Histological analysis

**FIGURE 1:**
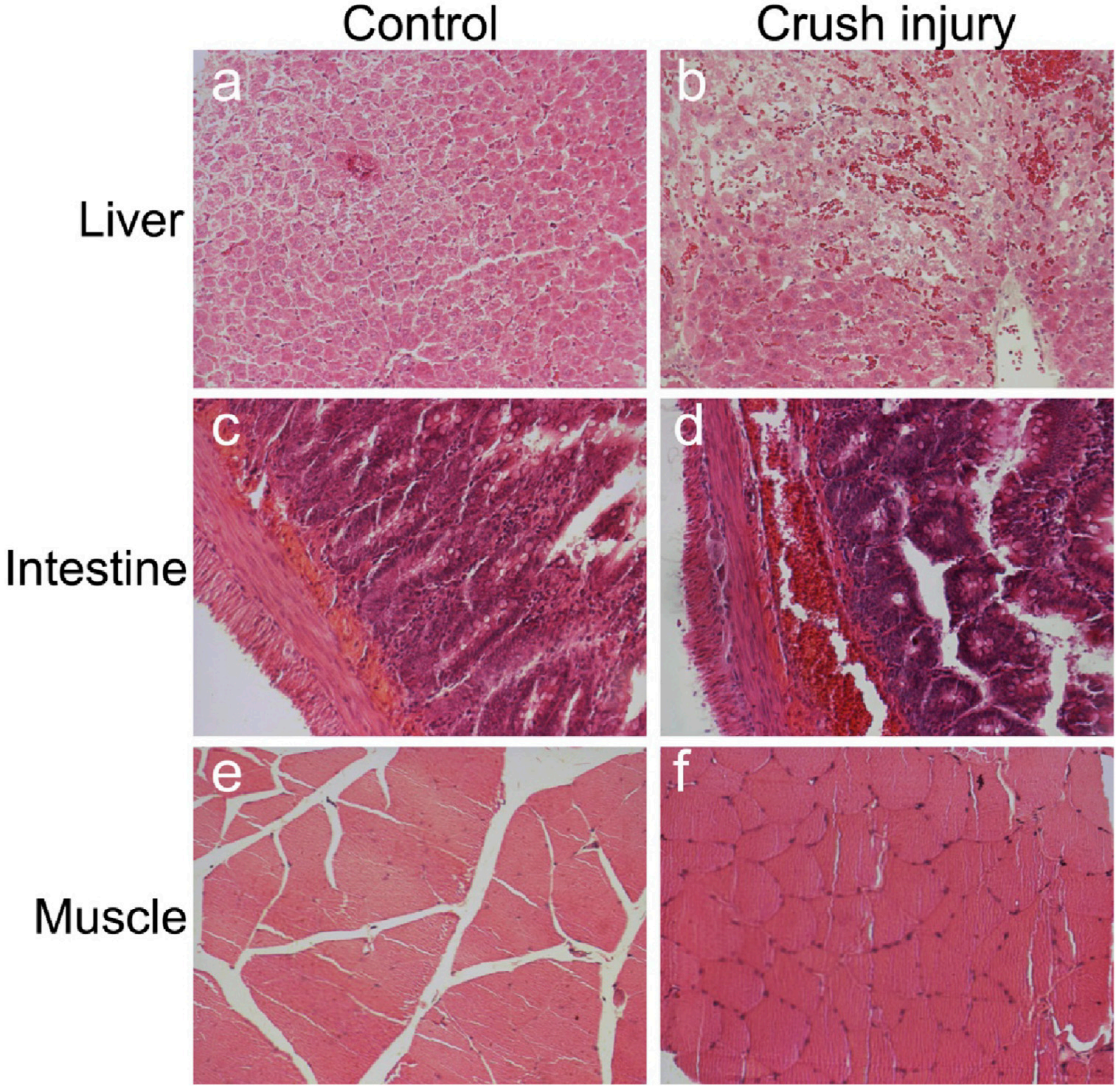
Observing Liver, Intestinal, and Skeletal Muscle Changes Through HE Staining. Note: **(A)** Liver in the control group; **(B)** Liver in the multiple traumas group; **(C)** Intestines in the control group; **(D)** Intestines in the multiple traumas group; **(E)** Skeletal muscle in the control group; **(F)** Skeletal muscle in the multiple traumas group. (Magnification: 200x).

**FIGURE 2:**
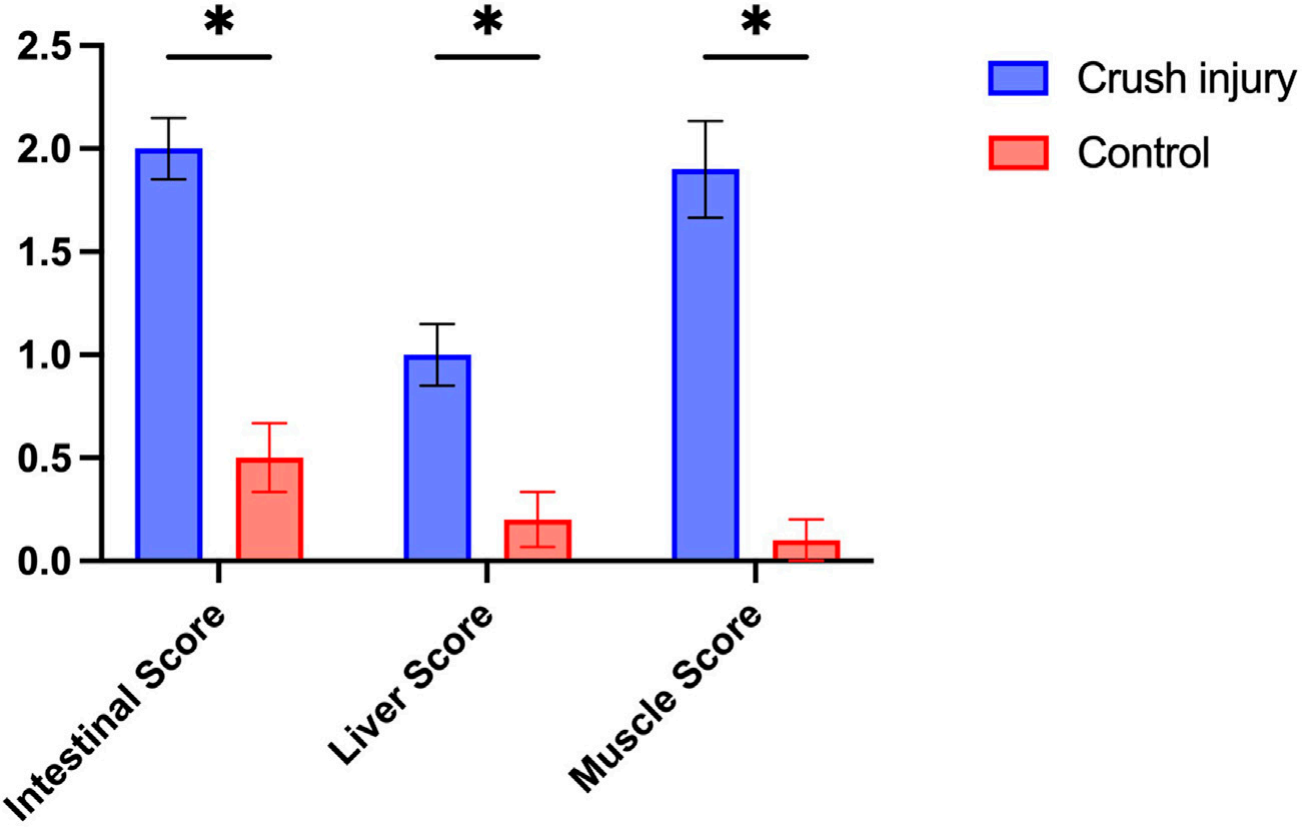
Pathological Scoring of Intestinal, Liver, and Skeletal Muscle Tissues in Rats from the Multiple Traumas and Control Groups. Note: The figure illustrates the pathological scoring of intestinal, liver, and skeletal muscle tissues in rats from the Multiple Traumas group (n = 10) and the Control group (n = 10). Each group’s scores are presented as mean ± standard error. The Multiple Traumas group scored significantly higher in all three tissue evaluations than the Control group (*P< 0.05).

This study conducted a detailed analysis of the tissue changes in rats after multiple traumas and shock through histological observations and X-ray examinations. Regarding liver tissue, the control group demonstrated intact structures of hepatic lobules, hepatic cords, hepatic sinuses, and portal areas (Figure 1A). Post-compression injury, mild swelling and hemorrhagic lesions were observed (Figure 1B). According to Suzuki et al.'s liver injury scoring system, the results showed a significantly higher liver injury score in the multiple traumas group compared to the control group (P< 0.01) (Figure 2). The control group scored (0.1 ± 0.32) points, while the multiple traumas group scored (1.7 ± 0.48) points, indicating notable structural changes in the liver tissue of the multiple traumas group without extensive necrosis.

For the intestinal tissue in the control group, the intestinal mucosa and glands were closely arranged without any signs of bleeding, erosion, or damage (Figure 1C). However, post-compression injury, the villous structure of the intestines was disrupted, the mucosal structure was disturbed, and extensive red blood cell infiltration was observed in the submucosal layer (Figure 1D). The results of the intestinal pathological changes scoring showed that the uninjured control group scored (0.3 ± 0.48) points, while the multiple traumas group scored significantly higher at (2.4 ± 0.52) points, surpassing the control group (P< 0.01) (Figure 2).

Regarding skeletal muscle tissue, the control group exhibited orderly arranged muscle fibers (Figure 1E), whereas post-compression injury, muscle cell swelling occurred without observed necrosis or hemorrhagic lesions (Figure 1F). The results of the skeletal muscle pathological damage scoring showed that the multiple traumas group had significantly higher muscle damage scores compared to the control group (P< 0.01) (Figure 2). Specifically, the control group scored (0.1 ± 0.66) points, while the multiple traumas group scored (2.6 ± 0.52) points, suggesting muscle cell nuclear shrinkage and <20% muscle cell necrosis induced by multiple traumas.

These findings indicate significant structural changes in the intestinal, liver, and skeletal muscle tissues of rats following multiple traumas and shock, although these changes did not lead to extensive necrosis or hemorrhagic lesions.

### Changes in vascular structure in the liver and intestine after injury

Understanding the integrity of blood vessel walls and their alterations post-injury is crucial in elucidating the pathophysiological processes in tissue damage research. In this study, Masson’s trichrome staining was employed to comprehensively observe the vascular walls and hemorrhage in liver and intestinal tissue samples from the control and compression injury groups.

**FIGURE 3:**
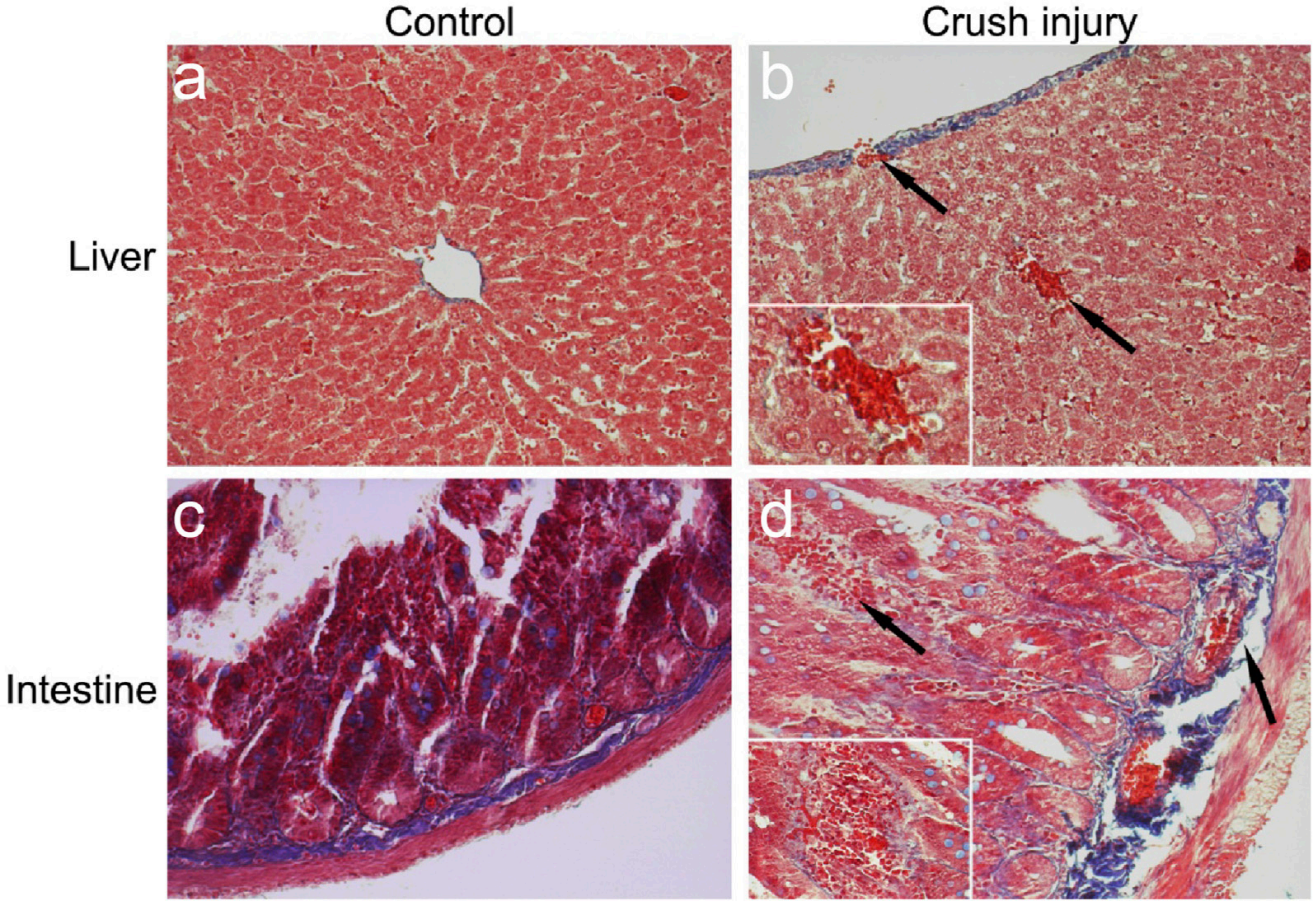
Masson’s Trichrome Staining Observations of the Liver and Intestine in a TIC Model. Note: **(A)** Liver in the control group: Shows normal tissue structure with intact collagen fibers (magnification: 200x). **(B)** Liver in the multiple traumas group: Displays ruptured collagen fibers and local hemorrhage, with arrows indicating the injured area. The inset provides a detailed image of the local hemorrhage (magnification: 400x), reflecting significant tissue structural changes post-injury. **(C)** Intestine in the control group: Demonstrates orderly tissue structure with well-arranged collagen fibers (magnification: 200x). **(D)** Intestine in the multiple traumas group: Exhibits ruptured collagen fibers and local hemorrhage, with arrows indicating the injured region. The inset offers a detailed image of the local hemorrhage (magnification: 400x), revealing tissue structural damage caused by trauma.

Figure 3 presents the microscopic observations of liver and intestinal tissues from the control group and those subjected to compression injury. In the control group’s liver and intestinal sections, blood vessel walls exhibited a regular layered arrangement and intact collagen fibers. In contrast, the vascular walls of the compression group tissues displayed significant structural disarray with ruptured collagen fibers. Furthermore, a significant infiltration of red blood cells was observed in the injured tissues, indicating hemorrhage occurrence.

These findings suggest a substantial disruption in vascular structure in the liver and intestine after injury, which may impact tissue blood supply and repair capacity, exacerbating local inflammatory responses.

### Radiological observation of tibia and fibula fractures and soft tissue swelling in a TIC model

**FIGURE 4:**
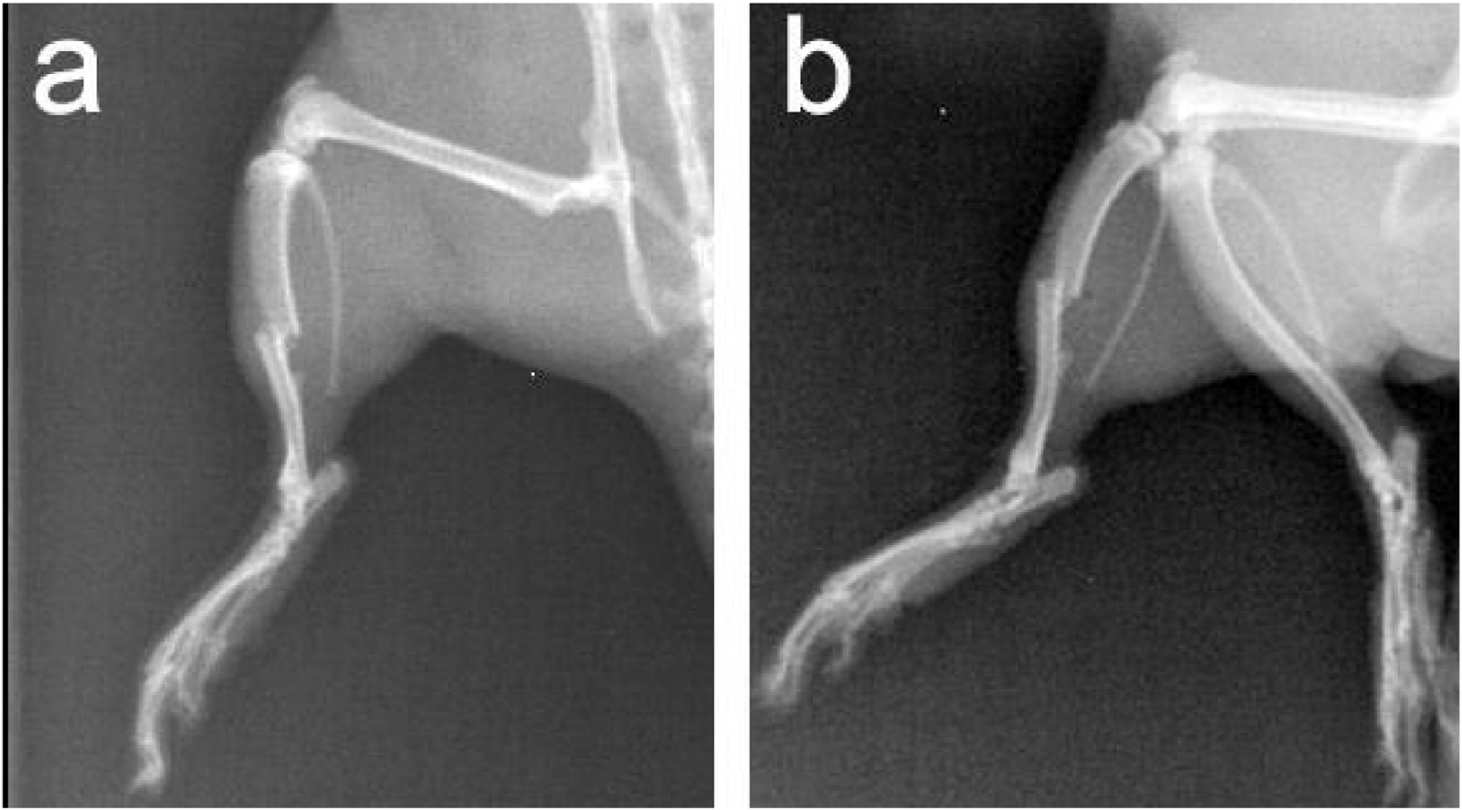
Anteroposterior and Lateral X-Ray Images of Tibia and Fibula in Rats with Multiple Traumas and Hemorrhagic Shock. Note: **(A)** Anteroposterior X-ray image; **(B)** Lateral X-ray image.

While investigating the TIC rat model, common clinical scenarios of multiple traumas were simulated through a series of trauma and hemorrhagic shock procedures. Routine radiological methods were used in this study to observe tibia and fibula fractures in rats. The results revealed that in rats subjected to multiple traumas and hemorrhagic shock, not only were fractures of the tibia and fibula prominently observed, but also significant local soft tissue swelling (Figure 4). These radiological findings were consistent with changes in other coagulation function parameters in the experiments, such as a marked elongation of APTT and a significant decrease in PLT count. These radiological discoveries further validate the significant impact of trauma and bleeding on coagulation function, emphasizing their crucial role in the TIC model.

### Temporal changes in coagulation function, inflammatory response, endothelial injury, and physiological indicators in a multiple traumas rat Model

**FIGURE 5:**
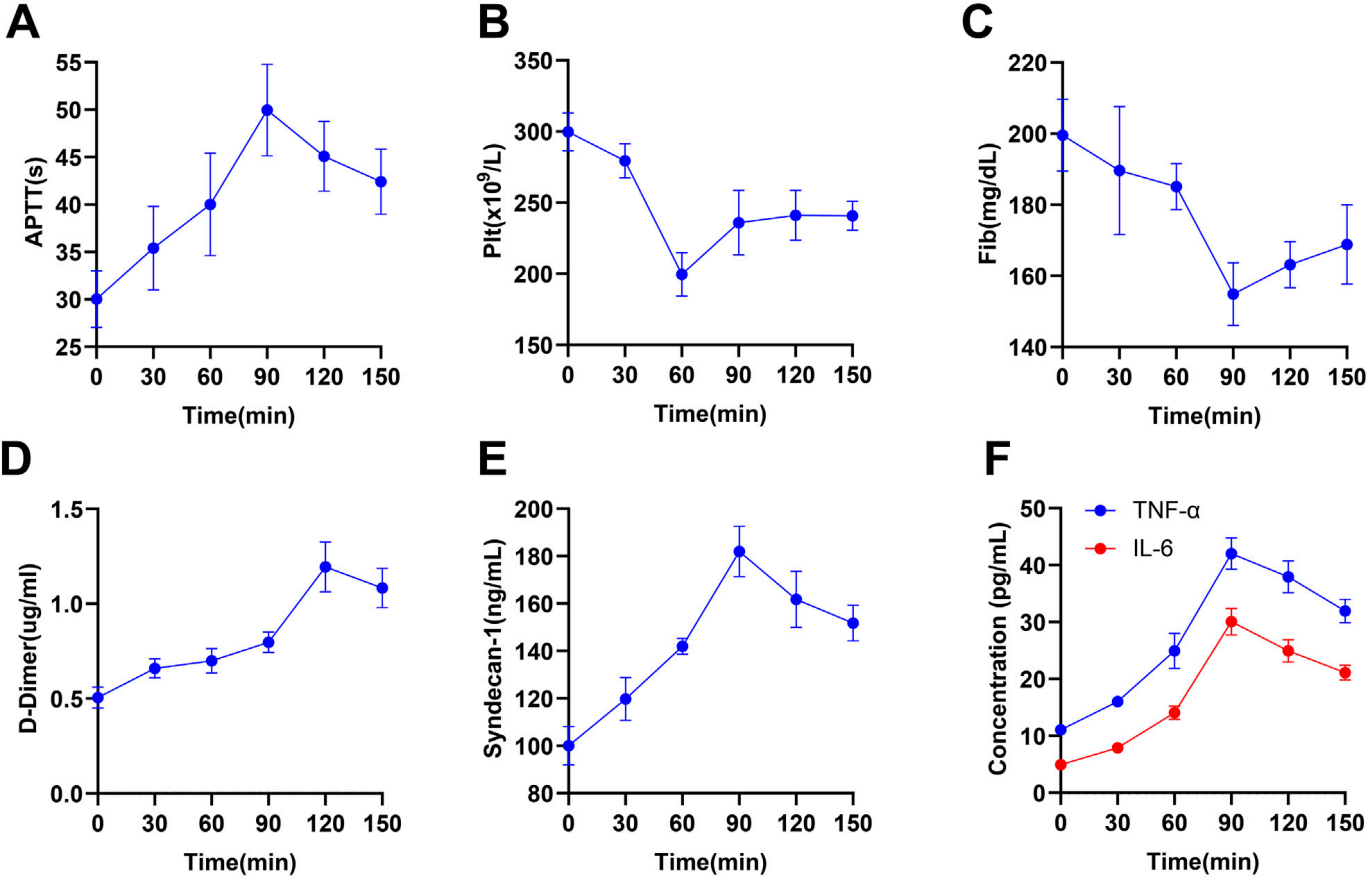
Temporal Changes in Coagulation, Endothelial, and Inflammatory Marker Levels in a Multiple Traumas Rat Model. Note: The figure displays data on the levels of coagulation, endothelial, and inflammatory markers in rats from the multiple traumas group (n = 10) at different time points (0, 30, 60, 90, 120, 150 min), with each level represented by the mean ± standard error. Specifically, it includes **(A)** APTT, **(B)** PLT count, **(C)** Fib, **(D)** D-dimer levels, **(E)** Syndecan-1, **(F)** inflammatory markers (TNF-α and IL-6).

In the study of TIC, precise monitoring of levels of coagulation function, inflammatory response, endothelial injury, and physiological indicators is crucial for understanding the dynamic processes of TIC. This study conducted Wilcoxon signed-rank tests to analyze these significant indicators individually, revealing key pathological changes in TIC (Table 2). Firstly, APTT displayed significant changes at multiple time points, notably reaching significant prolongation at 90 min (Figure 5A, P< 0.05), suggesting a notable impact on coagulation function at this time point. Concurrently, PLT count showed a significant decrease at 60 min (Figure 5B, P< 0.05), indicating that platelets may have been consumed or aggregated during the coagulation process following trauma, reflecting an increased demand for coagulation.

**TABLE 2:** Time-dependent changes in coagulation and physiological parameters: one-sided paired wilcox test results.

	30min	60min	90min	120min	150min
APTT	2.67E-04	5.34E-05	3.81E-06	1.91E-05	1.40E-03
PT	-	-	-	-	-
INR	-	-	-	-	-
Plt	1.91E-05	1.14E-05	1.07E-04	1.14E-05	3.81E-06
Fibrinogen (Fib)	1.30E-01	8.00E-02	5.00E-02	7.00E-02	1.00E-01
D-dimer	6.00E-02	4.00E-02	3.00E-02	1.00E-03	5.00E-02
TNF-α	7.00E-02	3.00E-02	1.00E-02	4.00E-02	6.00E-02
IL-6	6.00E-02	4.00E-02	1.00E-02	5.00E-02	7.00E-02
Syndecan-1	8.00E-02	5.00E-02	5.00E-02	6.00E-02	7.00E-02
Breath rate	3.22E-02	2.44E-02	1.61E-02	1.95E-03	9.77E-04
Blood pressure	9.77E-04	9.77E-04	1.95E-03	9.77E-04	9.77E-04

Fib significantly decreased to its lowest point at 90 min (Figure 5C, P< 0.05), followed by a slight recovery but not fully restored by 150 min, reflecting consumption of clotting factors and impairment of coagulation function. D-dimer peaked at 120 min (Figure 5D, P< 0.001), indicating increased thrombus formation and fibrinolytic activity. Additionally, Syndecan-1, a marker of endothelial injury and increased vascular permeability, significantly increased at 90 min (Figure 5E, P< 0.05), further confirming endothelial barrier dysfunction. Inflammatory markers TNF-α and IL-6 began significantly rising at 30 min, peaking at 90 min (Figure 5F, P< 0.01), reflecting acute inflammatory response and activation of immune cells triggered by trauma. Significant changes in APTT during 30–60 min and 90–120 min in Figure 5 remind us to focus on coagulation monitoring in the early stages of trauma. The significant drop in PLT count during 30–60 min further emphasizes this period as a potential key point for clinical intervention. Respiratory rate showed significant changes from 60 min onward until the end of the experiment, while significant variations in blood pressure from 30–90 min suggested hemodynamic instability during this period (Table 3).

**TABLE 3:** Temporal variations in coagulation and physiological parameters: one-sided paired wilcox test results across different time intervals.

	30–60min	60–90min	90–120min	120–150min
APTT	0.02	0.47	0.05	0.96
Plt	0.43	0.00	0.43	0.29
Breath rate	0.27	0.04	0.04	0.03
Blood pressure	0.35	0.04	0.46	0.08

**FIGURE 6:**
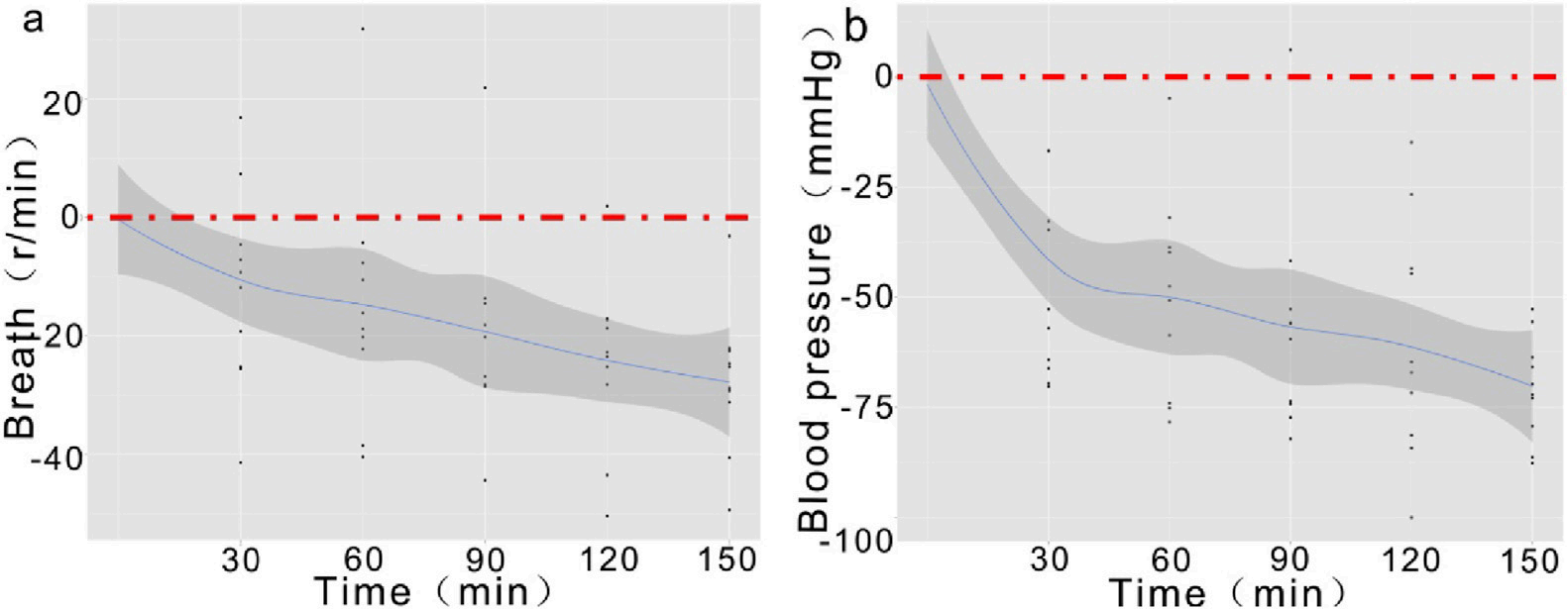
Temporal Trends of Respiratory Rate and Blood Pressure Post-Trauma. Note: **(A)** Demonstrates a gradual decrease in respiratory rate starting at 30 min, with a more pronounced decline by 150 min; **(B)** The declining trend in blood pressure initiates at 30 min and persists until 150 min, showing an overall significant decrease.

The continuous monitoring of respiratory rate and blood pressure further revealed the gradual deterioration of physiological function post-trauma. These indicators presented time-dependent significant changes at all time points, showing a consistent declining trend (Figure 6), aligning with the expected physiological response post-trauma.

The statistical analysis results of this study demonstrate time-dependent significant decreases in respiratory rate and blood pressure in the TIC model.

### Correlation between PLT count and inflammatory markers in a rat model following multiple traumas

**FIGURE 7:**
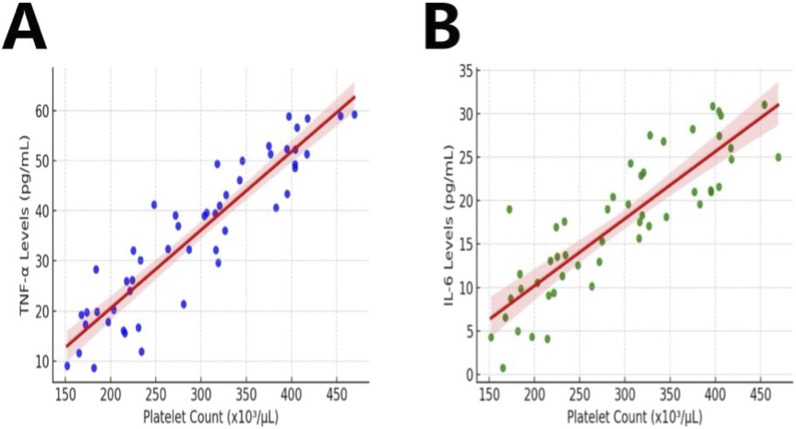
Correlation Between PLT Count and Inflammatory Markers in Rats with Multiple Traumas. Note: The figure depicts data on PLT count and levels of inflammatory markers in rats (n = 10) in the Multiple Traumas group during the observation period. Figures **(A, B)** illustrate the correlation between PLT count and TNF-α, IL-6.

In this study, we comprehensively investigated the correlation between PLT count and the inflammatory factors TNF-α and IL-6 in a rat model post multiple traumas using Spearman’s rank correlation analysis. The results indicated a significant positive correlation between the decrease in PLT count and the increase in the concentrations of TNF-α and IL-6, suggesting a strong positive relationship between them (Figures 7A, B; ρ > 0.5, *p* < 0.05). This finding indicates a significant association between inflammation activation and platelet changes; however, it is insufficient to infer a clear causal relationship.

## Discussion

**FIGURE 8:**
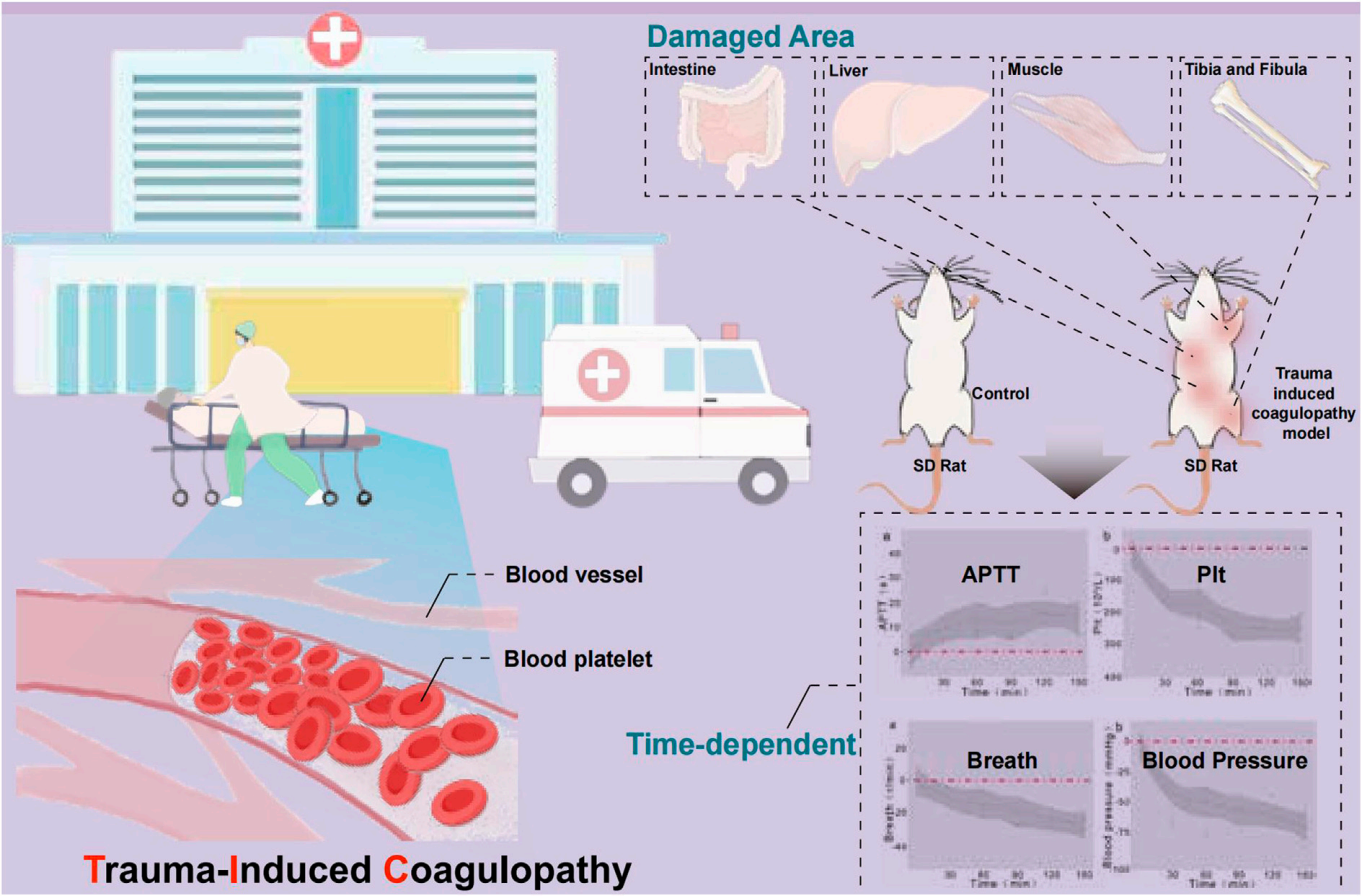
Early coagulation dynamics in a rat model of polytrauma and hemorrhagic shock.

TIC, as a common complication of multiple trauma, involves a complex pathophysiology encompassing vascular endothelial damage, inflammatory response, and coagulation dysfunction. This study established a rat model of multiple traumas and shock, revealing elongated APTT and decreased PLT count, consistent with clinical manifestations in TIC patients. These findings offer a new perspective for understanding the pathogenesis of TIC (Figure 8).

Compared to prior studies, this research introduced methodological innovations. Demonstrated in this study is an effective femoral artery cannulation technique, enabling stable continuous blood sampling in a single rat model of TIC for intermittent coagulation parameter measurements. Longitudinal sampling over a short period is challenging in clinical settings, making animal models valuable for reliable TIC investigations. Various TIC experimental models have been used in recent years. The rat model of TIC described by Daniel and colleagues in 2013, representing TIC and hemorrhagic shock, has been notable, categorizing rats into different groups at 30, 60,120,180, and 240 min post-bleeding initiation. However, a control group was not included. Blood samples were obtained at point 0, serving as self-control in our work. Additionally, our study introduced a control group where the femoral and carotid arteries were cannulated but without trauma or bleeding.

Clinical data indicate that TIC is most prevalent in severely injured patients with extensive tissue damage and hemorrhagic shock (Mowbray et al., 2021; Lainé et al., 2022; Frith et al., 2010). Furthermore, HE staining in our work confirmed tissue damage. Masson’s staining revealed damaged microvessel walls in the liver and small intestine post-crush injury, including collagen fiber fragmentation and endothelial injury. Involvement of neurotransmitters and endothelial response in acute hemorrhage contributing to TIC development has been reported (Diebel et al., 2018; Johansson et al., 2012). Though the precise mechanisms behind shock-induced endothelial activation and dysfunction remain unclear, evidence increasingly suggests a crucial role of endothelial barrier disruption in the endothelial response to traumatic shock (Haywood-Watson et al., 2011), potentially associated with APTT elongation (Ke et al., 2022; Johansson et al., 2011). Therefore, our damaged vessels may promote coagulopathy development. APTT is used to diagnose coagulopathies like TIC (Ellervik et al., 2021; Brohi et al., 2007; Brohi et al., 2008; Frith et al., 2010). Significant deviations in APTT and PLT count compared to the control group suggest the presence of coagulopathy (Almeida et al., 2021; Zeng and Liao, 2022; Shaheen et al., 2020). Moreover, we observed substantial APTT changes between 30–60 min and 90–120 min compared to the control group, indicating an early rise in APTT post-injury. It finding parallels clinical observations in patients (Floccard et al., 2012), suggesting the occurrence of TIC in the pre-hospital phase before extensive fluid resuscitation (Buzzard and Schreiber, 2024; Horst et al., 2020; Wafaisade et al., 2010; Wohlauer et al., 2012). Tissue damage and shock appear to be primary drivers of early coagulopathy post-trauma. Contrary to prior studies, the PT in the multiple trauma group rats remained normal, implying that the extrinsic coagulation pathway may not be immediately activated in our model, warranting further investigation.

Currently, mounting evidence supports the pivotal role of PLT dysfunction in the pathophysiology of TIC (John and White, 2020; Dobson et al., 2020; Frith et al., 2010). The quantity of PLTs in circulation strongly influences hemostasis (Raghunathan et al., 2022; Althaus et al., 2021; Singh et al., 2021). Decreased PLT count in trauma patients can predict higher mortality rates (Connelly et al., 2017; Stansbury et al., 2013). Our TIC rat model observed a significant reduction in PLT count, likely attributable to hemodilution and consumption post-blood volume restoration. PLT count rose close to baseline at 60 min, possibly due to release from reserves (spleen or bone marrow) or new megakaryocyte formation (Lazar and Goldfinger, 2021; Tao et al., 2021; Darlington et al., 2013).

The results of this study have significant clinical application potential in the early diagnosis and intervention of TIC. By observing the time-dependent relationship between APTT prolongation, platelet count reduction, and inflammatory markers such as TNF-α and IL-6 in a rat model, we provide new biomarkers for the early clinical identification of TIC. This discovery is particularly important for the management of trauma patients in the fields of emergency and critical care medicine. Through structured early monitoring, these biomarkers can guide targeted treatments, helping clinicians intervene effectively during the critical post-trauma window, thereby reducing the risk of complications and mortality. Additionally, the findings of this study can be extended to other types of trauma patients, particularly in cases involving multiple traumas or significant blood loss, offering broad application prospects.

The value of this study lies in the first systematic analysis of the correlation between tissue structural changes and coagulation dysfunction in a rat TIC model, providing new insights into the pathogenesis of TIC. These findings not only help reveal TIC’s pathophysiological processes but also provide scientific evidence for developing new diagnostic and therapeutic strategies in clinical practice. By identifying and intervening with these biomarkers early, clinicians can implement focused interventions within 90 min after trauma, improving treatment outcomes and patient prognosis.

Future studies should validate the effectiveness of these biomarkers in human clinical trials to facilitate the translation of these findings into clinical practice. By combining emerging anti-inflammatory treatments and coagulation factor supplementation strategies, TIC management protocols can be further optimized, increasing the precision and personalization of treatments. Furthermore, with the continuous improvement of monitoring and diagnostic techniques, these discoveries can be widely applied to different types of trauma and shock patients, advancing the clinical management of TIC from theory to practice.

While this study yielded valuable data in a Multiple Trauma Rat Model, it has certain limitations. Firstly, animal models cannot entirely replicate human physiological and pathological processes; thus, caution is needed in generalizing the research findings. Notably, rats’ coagulation and inflammatory responses may differ significantly from those in humans, which requires further validation when translating these results into clinical practice. Secondly, the study did not encompass all potential biomarkers influencing TIC nor consider the potential impact of genetic and environmental factors on TIC development. Additionally, the sample size utilized in the study may limit the statistical power of the results. Future research should aim to expand the sample size, incorporate a broader array of biomarkers, and consider the diversity of genetic backgrounds and environmental factors.

Future research should focus on exploring additional biomarkers, especially novel ones related to coagulation function and inflammatory responses, to improve the accuracy of early TIC diagnosis. Furthermore, future studies should validate the findings of this study in clinical trials to ensure the applicability and effectiveness of these biomarkers in human patients. Developing new intervention strategies, particularly for early interventions within specific time windows (such as monitoring platelet function and administering anti-inflammatory treatments within the first 90 min), will also be a key focus of future research.

Moreover, research efforts should focus on verifying whether the findings from the rat model can be translated for clinical application. Further studies are required to develop new strategies for early TIC intervention and evaluate the effectiveness of these strategies in clinical trials. Ultimately, this study provides a solid starting point for TIC’s future foundational and clinical research.

In summary, this study meticulously observed post-traumatic tissue damage and analyzed the changes in coagulation function and physiological indicators by establishing a Multiple Trauma and Shock Rat Model. The results indicate that multiple trauma and shock could successfully induce TIC in the rat model, with observed time-dependent elongation of APTT, decreased PLT count, and a decline in respiratory rate and blood pressure. These changes offer new biomarkers and potential therapeutic windows for early diagnosis and treatment of TIC.

## Data Availability

The original contributions presented in the study are included in the article/Supplementary Material, further inquiries can be directed to the corresponding author.

## Supplementary material

SUPPLEMENTARY FIGURE S1 Observation of Tissue Damage after Multiple Traumas and Shock in Experimental Rats.
