# Supplementary figures and images for: Correlations between coagulation abnormalities and inflammatory markers in trauma-induced coagulopathy
Source: Front Physiol. 2024 Oct 30;15:1474707. doi: 10.3389/fphys.2024.1474707 (PMC11557354; doi:10.3389/fphys.2024.1474707)

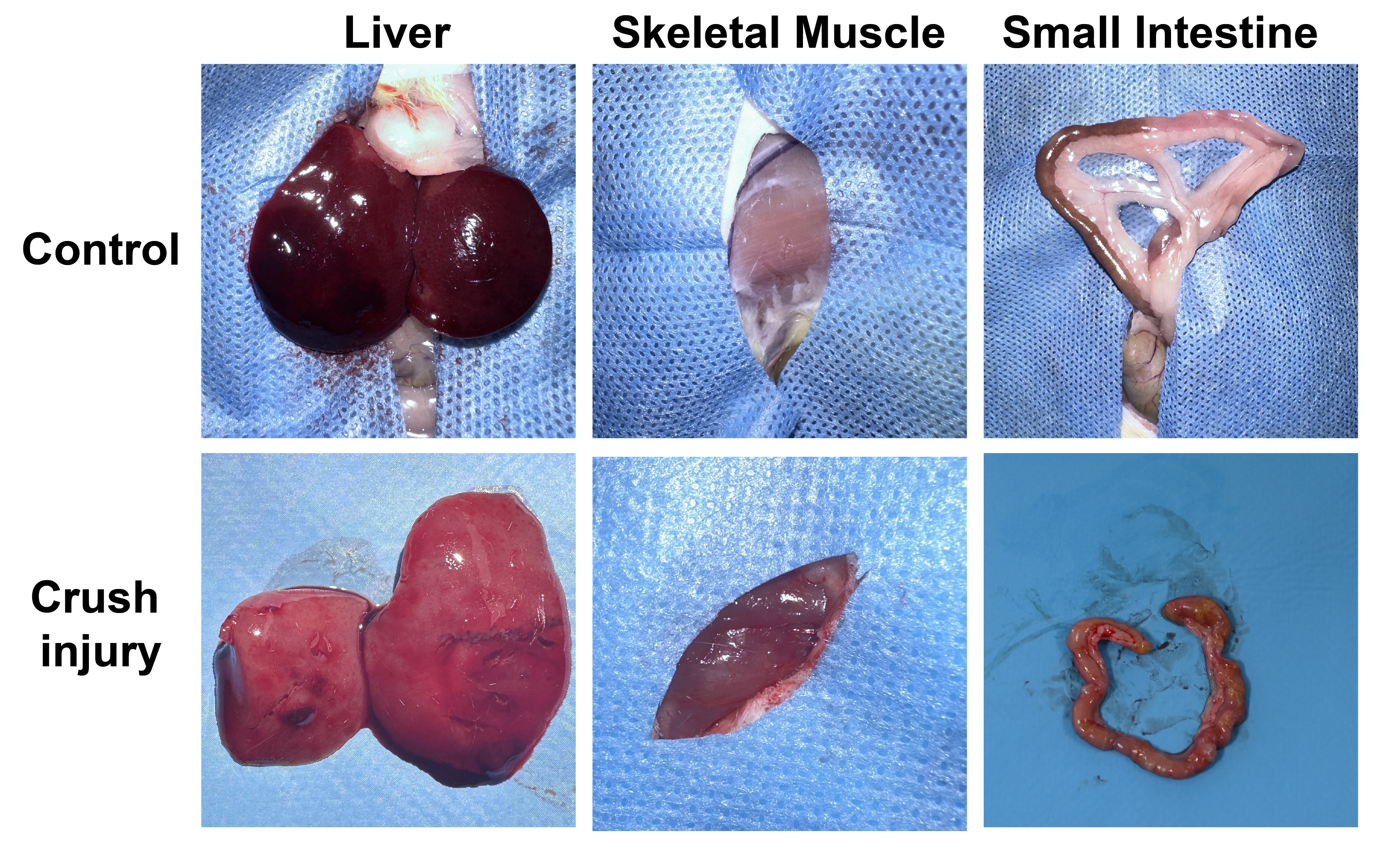

Supplement: Supplementary file 1 [file Image1.JPEG]
